# Cost-effectiveness of de-escalated molecular subtype dependent use of neoadjuvant chemotherapy in patients with muscle-invasive bladder cancer in a Swedish setting

**DOI:** 10.3389/fonc.2025.1556881

**Published:** 2025-04-02

**Authors:** Sanjib Saha, Ulf-Göran Gerdtham, Gottfrid Sjödahl, Christel Häggström, James W. F. Catto, John D. Kelly, Anders Ullén, Lars Holmberg, Fredrik Liedberg

**Affiliations:** ^1^ Health Economics Unit, Department of Clinical Sciences (Malmö), Lund University, Lund, Sweden; ^2^ Department of Economics, Lund University, Lund, Sweden; ^3^ Division of Clinical and Experimental Urothelial Carcinoma Research, Department of Translational Medicine, Lund University, Malmö, Sweden; ^4^ Department of Urology, Skåne University Hospital, Malmö, Sweden; ^5^ Department of Surgical Sciences, Uppsala University, Uppsala, Sweden; ^6^ Northern Registry Centre, Department of Diagnostic and Intervention, Umeå University, Umeå, Sweden; ^7^ Division of Clinical Medicine, School of Medicine & Population Health, University of Sheffield, Sheffield, United Kingdom; ^8^ Division of Surgery & Interventional Science, University College London, London, United Kingdom; ^9^ Department of Oncology-Pathology, Karolinska Institute, Stockholm, Sweden; ^10^ Department of Pelvic Cancer, Genitourinary Oncology and Urology Unit, Karolinska University Hospital, Stockholm, Sweden; ^11^ Division of Cancer Studies, Medical School, King’s College London, London, United Kingdom

**Keywords:** cost-effectiveness analysis, muscle invasive bladder cancer, molecular subtype, neoadjuvant chemotherapy, radical cystectomy

## Abstract

**Background:**

Guidelines recommend neoadjuvant chemotherapy (NAC) and radical cystectomy (RC) for muscle-invasive bladder cancer (MIBC). Current recommendations do not consider genomic profiles, although the Basal/Squamous (Ba/Sq) subtype is less likely to respond to NAC compared to Urothelial-like (Uro) and Genomically Unstable (GU) subtypes. The aim of this study is to perform cost-effectiveness analyses of a de-escalated use of NAC in patients with Ba/Sq tumors and MIBC.

**Methods:**

A cost-effectiveness analysis was performed using a decision analytic Markov model using a healthcare provider perspective. Treatment and prognosis probabilities originated from the Bladder Cancer Data Base, Sweden (BladderBaSe) 2.0. Information on molecular subtype and outcomes was retrieved from published studies, and quality-adjusted life year (QALY) data were obtained from the iROC trial. Costs were collected from the regional healthcare registers in Sweden, utility values were obtained from the literature, and outcomes are presented as incremental cost-effectiveness ratio (ICER). Scenario analyses, along with several one-way and probabilistic sensitivity analyses were performed to capture uncertainties.

**Results:**

At a 5-year time horizon, the model predicts that molecular subtype-based treatment has an ICER of 4,964 Euro/QALY (66,766 Swedish Krona/QALY), which is deemed cost-effective in the Swedish setting. At €7,427 (100,000 SEK) willingness-to-pay threshold, the molecular subtype-based treatment has a 65% probability of being cost-effective. The results were not sensitive to uncertainty analyses.

**Conclusion:**

Molecular subtype-based treatment of MIBC, i.e., refraining from administering NAC to patients with Ba/Sq tumors, is cost-effective compared to the current treatment practices in Sweden.

## 1 Introduction

With the advent of molecular classification based on transcriptomic profiling (1) and subsequent studies suggesting a role for molecular subtyping in predicting neoadjuvant chemotherapy (NAC) response (2–4), possibilities to individually tailor the use of NAC have emerged. By applying the Lund taxonomy (LundTax), we have shown that patients with the Basal/Squamous (Ba/Sq) subtype are less likely to benefit from three courses of cisplatin-based chemotherapy compared to those with Urothelial-like (Uro) or Genomically Unstable (GU) subtypes, both when evaluating the pathological response in the cystectomy specimen and the survival outcomes (3).

Today, the standard of care for all patients with muscle-invasive bladder cancer (MIBC) without metastases (T2-T4aN0M0) is cisplatin-based NAC followed by radical cystectomy (RC). The use of NAC gains a 5% absolute survival benefit at five years compared to RC only (5). In Sweden, 113 out of 152 (74%) patients under 76 years received such preoperative treatment in 2022 (6). For patients not receiving NAC, cisplatin-based chemotherapy can be considered in the adjuvant setting for selected patients with advanced disease (pT3/pT4 and/or N+) according to Swedish and EAU guidelines (7).

Applying a molecular subtype-based and de-escalated use of NAC to reduce overtreatment combined with offering adjuvant chemotherapy only to those with advanced disease in the cystectomy specimen may entail a better utilization of healthcare resources. It might also increase the uptake of NAC, which up to now have had a low utilization rate of only 17.2% according to a recent meta-analysis (8). This is particularly important considering that bladder cancer is one of the most expensive malignancies, with a total annual cost of around $7.93 billion in the USA (in 2015) and €5.24 billion in Europe (in 2019) (9).

To alter the current treatment practice, offering NAC to all eligible patients, and provide treatment according to molecular subtypes in line with the ideas behind precision medicine, a broad and comprehensive assessment including both effectiveness of treatment patterns as well as cost-effectiveness is needed. The objective of this study is to estimate whether molecular subtype-specific use of NAC in patients with MIBC is cost-effective compared to current practice in Sweden by applying a de-escalated use of NAC for the Ba/Sq subtype.

## 2 Materials and methods

In the absence of a clinical trial, this cost-effectiveness analysis utilized a Markov decision analytic simulation model following the Consolidated Health Economic Evaluation Reporting Standards (CHEERS) (10).

### 2.1 Study population

**Figure 1:**
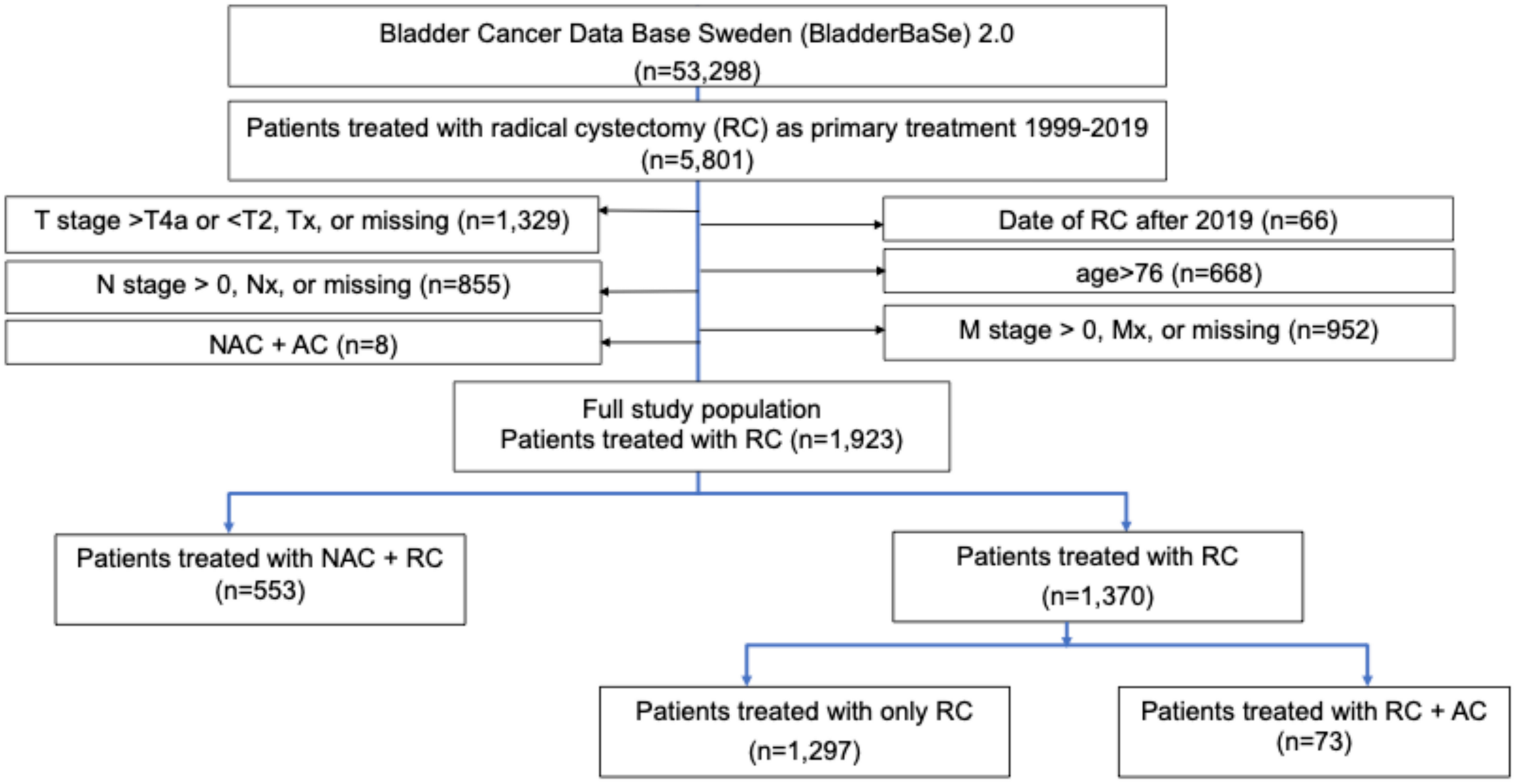
Study sample selection procedure from the BladderBase 2.0 database. RC, Radical Cystectomy; NAC, Neoadjuvant Chemotherapy; AC, Adjuvant Chemotherapy.

We used data from the Bladder Cancer Data Base Sweden (BladderBaSe 2.0), a research database including all patients in the Swedish National Register for Urinary Bladder Cancer (SNRUBC) diagnosed from the 1^st^ of January 1997 through the 31^st^ of December 2019, individually linked by using the unique Swedish personal identification number to several Swedish national data sources (11). BladderBaSe 2.0 includes information on tumor characteristics, treatments, and important confounding factors such as socioeconomic variables and comorbidity, and also with extensive follow-up. As a proxy for cisplatin-eligibility, we choose from this database all patients who were 76 years or younger at the time of RC as their main treatment for clinical stage T2-T4aN0M0 disease from 1999 to 2019. This is also in line with the Swedish national guidelines on urothelial carcinoma recommending that above 60% in this population should be offered NAC (12). We excluded patients who were treated with both NAC and adjuvant chemotherapy (AC), given that this is not recommended by EAU guidelines (7). The study population sample selection procedure is displayed in **Figure 1**.

### 2.2 Markov model structure

We created a Markov decision-analytic simulation model comparing current treatment practice when offering NAC to all cisplatin-eligible patients with MIBC to a de-escalated use of NAC based on molecular subtype. In the current treatment practice, patients receive either three courses of NAC, with ddMVAC (methotrexate, vinblastine, Adriamycin, and cisplatin), followed by RC (NAC+RC) or RC only. Those who received upfront RC without NAC optionally received four courses of AC (ddMVAC) in cases of non-organ-confined disease in the cystectomy specimen (i.e., pT3 or higher or pN+).

**Figure 2:**
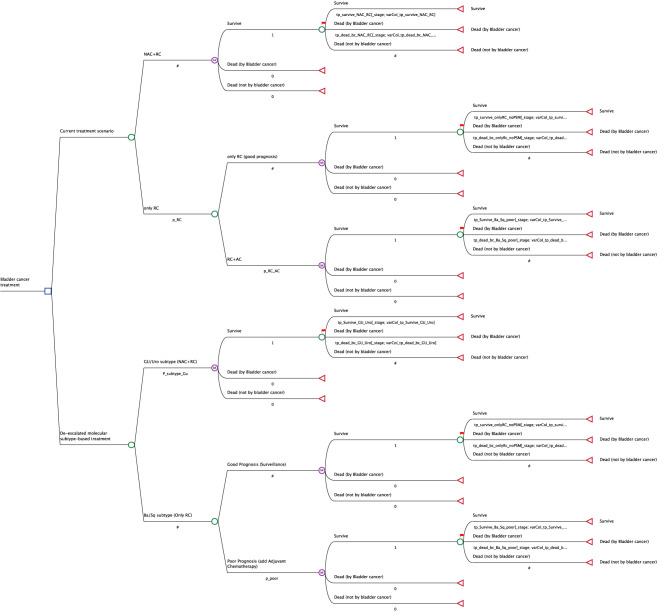
The Markov model structure.

In the molecular subtype-based treatment option, patients with either Uro or GU tumors will receive NAC+RC, while those with the Ba/Sq subtype will receive upfront RC, but if the cystectomy specimen displays pT3 or higher or pN+, four courses of AC will be administered postoperatively according to guidelines. The Markov model is based on only three health states: survival after RC, death by bladder cancer, and death by other causes than bladder cancer, where patients transition into these health states are estimated in a yearly cycle. In **Figure 2**, the Markov model structure is described. The model is developed and analyzed using TreeAgePro Healthcare, v2023.

### 2.3 Proportion and probabilities

The proportion of patients receiving NAC+RC, upfront RC only, and RC+AC was estimated by use of BladderBaSe 2.0. Since BladderBaSe 2.0 does not have information on molecular subtype, the percentage of individuals with Uro/GU and Ba/Sq subtypes, respectively, was estimated using data from Sjödahl et al. (3) and Olah et al. (13). The proportion of patients with Ba/Sq subtypes who had poor prognosis (≥pT3 or pN+) based on inclusion criteria in adjuvant trials (14, 15) and the corresponding proportion of good prognosis (≤pT2N0) according to the pathological tumor stage in the cystectomy specimen after RC was also estimated from these two studies (3, 13). Assuming cisplatin eligibility before and after RC, the poor prognosis definition was applied to determine the proportion of patients treated with upfront RC who would be recommended for AC. The criteria for poor and good prognosis are presented in **Table 1**, and the parameters of the model are given in **Table 2**.

**Table 1:** The criteria for good and poor prognosis are based on the pathological tumour stage (pT-stage) and pathological nodal stage (pN-stage) in the radical cystectomy specimen.

	T stage	N stage
Good prognosis (A & B)	pT0, pTa, pTCIS, pT1, pT2, pT2a, pT2b, pTx, N/A	pN0, pNX, N/A
Poor prognosis (C or D)	pT3, pT3a, pT3b, pT4a, pT4b	pN1, pN2, pN3

**Table 2:** Cost (in Euro (EU-27), 2022 price year) and utility parameters used in the model.

Parameter	Point estimate	Range	Source
Probabilities			
Current treatment practice			
NAC+RC	0.280	0.252 to 0.740	BladderBaSe 2.0, expert opinion, (6)
Only RC	0.720	0.648 to 0.811	BladderBaSe 2.0, expert opinion, (16)
Poor prognosis	0.053	0.047 to 0.058	BladderBaSe 2.0, expert opinion
Good prognosis	0.947	0.853 to 0.99	BladderBaSe 2.0, expert opinion
Molecular subtype			
Uro/GU subtype (NAC+RC)	0.696	0.650 to 0.779	(3, 13)
Ba/Sq subtype (only RC)	0.303	0.220 to 0.349	(3, 13)
Poor prognosis	0.753	0.734 to 0.773	(3, 13)
Good prognosis	0.246	0.227 to 0.265	(3, 13)
Cost			
RC	14711	13240 to 17653	Region Skane
NAC (3 courses)	2270	2043 to 2724	TLV, Region Skane, Region Stockholm
AC (4 courses)	2586	2327 to 3103	TLV, Region Skane, Region Stockholm
Surveillance	444	400 to 533	Joyce et al. (17)
Identification of subtype	341	273 to 409	Region Skane
Utility			
Surveillance	0.914	0.8 to 1.0	iROC trial (18)
Cystectomy	0.80	0.6 to 1.0	Literature (17, 19)
Disutility NAC/AC	-0.36	-0.30 to -0.40	Literature (17, 19)

AC, Adjuvant chemotherapy; NAC, Neoadjuvant chemotherapy; RC, Radical Cystectomy; TLV, Dental and Pharmaceutical Benefits Agency in Sweden.

The yearly survival probabilities, including death due to bladder cancer or other causes, were retrieved from BladderBaSe 2.0 (n=1297). Additionally, we used data from Sjödahl et al. to estimate the survival for individuals with Uro/GU subtypes who received NAC+RC (n=77) (3). Due to a small sample size, survival probabilities for patients with Ba/Sq subtype in both the good and poor prognosis categories were uncertain. Instead, we used survival estimates from those treated with RC only (n=1297) and with RC+AC (n=73) in BladderBaSe 2.0, respectively (**Figure 1**). The yearly transition probabilities are presented in the **Supplementary Materials** (Section 1: **Supplementary Tables S1**-**S6**). The statistical analyses were conducted using STATA version 17.

### 2.4 Costs and utilities

This cost-effectiveness analysis was performed from a healthcare provider’s perspective. Costs due to RC, chemotherapy, and identification of molecular subtypes through RNA extraction and RNA-sequencing applying LundTax single sample classifiers (20) were estimated from the Skane Regional Council healthcare registers (**Table 2**). All healthcare services were valued by using diagnosis-related groups (DRG), a patient cost classification system (21). All costs were collected in Swedish kronor (SEK) and converted into Euro (27-EU) using purchasing power parity in the 2022 price year. (22). Utility values for the survival health states were obtained from the literature and expert opinion (**Table 2**). The utility values, toll, and time within each health condition, *i.e.*, RC with three or four courses of chemotherapy, were used to calculate quality-adjusted life years (QALYs) with the area under the curve methodology (23). Baseline QALY, *i.e.*, at the year of diagnosis, was obtained from the iROC trial (18). The assumptions behind the QALY estimations are presented in the **Supplementary Materials** (Section 2). Costs and QALYs were discounted at a rate of 3% annually following the guideline of the Dental and Pharmaceutical Benefits Agency (TLV) of Sweden (24).

### 2.5 Base case analyses of cost-effectiveness

The results were expressed as incremental cost-effectiveness ratio (ICER), where ICER is defined as the difference in costs divided by the differences in QALYs between groups. According to the Swedish National Board of Health and Welfare, cost per QALY gained below 100,000 SEK (7,427 Euro/QALY) is considered low cost-effective (25) and used as the willingness-to-pay (WTP) threshold in this study. The analysis applied a base case time horizon of two years, three years, and five years from the time of RC (**Table 3**).

**Table 3:** Incremental cost-effectiveness ratio (ICER) at 2-year, 3-year and 5-year by current treatment practice and de-escalated molecular subtype-based use of perioperative chemotherapy, respectively.

Time duration	Cost (Euro) (std)	Incremental cost	QALYs (std)	Incremental benefit	ICER (Euro/QALY)
2-year					
Current treatment	16,064 (1,175)		2.16 (0.65)		
Molecular subtype-	17,930 (711)	1,866	2.32 (0.55)	0.17	11,211
3-year					
Current treatment	16,245 (1,253)		2.53 (0.94)		
Molecular subtype-	18,160 (785)	1,914	2.80 (0.83)	0.27	7,192
5-year					
Current treatment	16,416 (1,387)		2.88 (1.34)		
Molecular subtype-	18,396 (939)	1,980	3.28 (1.29)	0.40	4,964

Std, Standard deviation.

### 2.6 Sensitivity and scenario analyses

Several one-way and probabilistic sensitivity analyses, together with three scenario analyses, were performed to estimate the uncertainty around the base case result. Sensitivity analyses were performed for the estimate using a 5-year time horizon. Most of the parameters in the model were varied over plausible ranges, and the outcomes were presented as a tornado diagram. For example, the proportion of patients receiving NAC in Sweden has increased over the years; thus, we used the most recent value from 2022 (74%) (6). Furthermore, we used the data from a recent German study on the proportion of upfront RC in a context where NAC, by tradition, is less frequently applied (16). For probabilistic sensitivity, Monte Carlo simulations with 10,000 iterations were employed and portrayed as Cost-Effectiveness Acceptability Curve (CEAC), which shows the probability of the intervention being cost-effective across a spectrum of WTP thresholds. The three scenario analyses are presented in the **Supplementary Materials** (Section 3).

## 3 Results

### 3.1 Base case analyses

In the base case analyses, the de-escalated molecular subtype-based treatment was associated with a 0.40 QALY increase compared to the current treatment practice with an additional cost of €1,980 at the 5-year time horizon. This yielded an ICER of €4,964/QALY, which is deemed cost-effective in the Swedish setting. With a WTP of 7,427 Euro, a de-escalated use of NAC was deemed cost-effective beyond a 2-year time horizon (**Table 2**).

### 3.2 Sensitivity analyses

**Figure 3:**
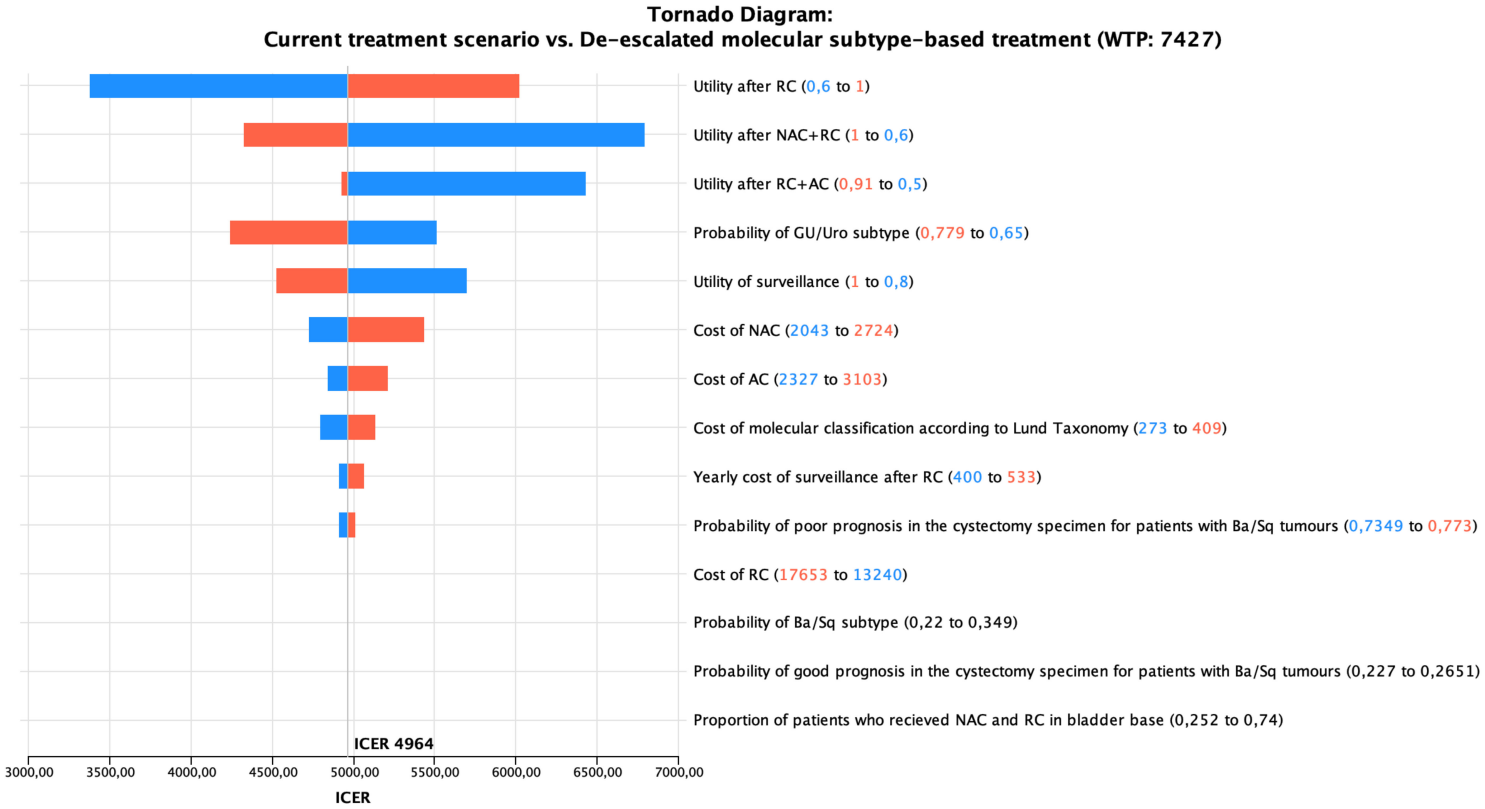
The Tornado diagram for one-way sensitivity analyses.

**Figure 4:**
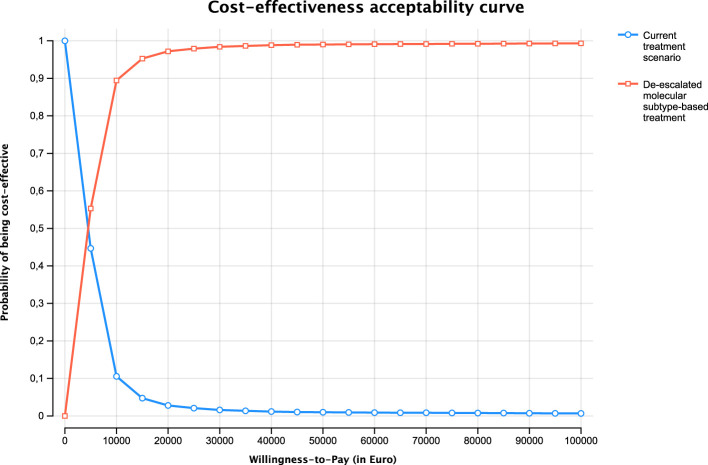
The cost-effectiveness acceptibility curve.

In the tornado diagram, while the base case result showed stability, the ICER demonstrated variability across parameters (**Figure 3**). The CEAC (**Figure 4**) showed that at €10,000 WTP, the molecular subtype-based treatment has a 90% probability of being cost-effective. The probability of being cost-effective increases with increasing WTP. The ICERs for the scenario analyses also fell below the WTP threshold except the third scenario (**Supplementary Materials**: Section 3 and **Supplementary Tables S8**-**S10**).

## 4 Discussion

We estimated the cost-effectiveness of molecular subtype-based use of NAC compared to the current treatment practice using population-based real-world data. We found that refraining from NAC in patients with Ba/Sq tumors and instead directly proceeding to upfront RC was cost-effective in base case, sensitivity, and scenario analyses.

To our knowledge, this is the first cost-effectiveness analysis using molecular subtype-based treatment for patients with MIBC. Thus, any comparisons with similar studies in bladder cancer were not possible. However, a de-escalated use of AC in breast cancer based on gene expression profiling has been reported as cost-effective (26), and so has molecular classification-based treatment in endometrial cancer (27).

The general lack of cost-effectiveness studies evaluating precision medicine in cancer was highlighted in a recent review, where the authors argued that the lack of clinical trials to estimate the effectiveness is a significant barrier to the implementation of targeted therapies (28). In the setting of de-escalating cancer treatment based on gene expression profiling, such lack of evidence is even more pronounced except for one large non-inferiority breast cancer RCT (n=10,273) (29). Due to the sample-size requirement, it is unlikely that an adequately designed non-inferiority RCT based on the 5% absolute survival benefit from NAC (5) investigating molecular subtype-dependent de-escalated use of NAC in bladder cancer will be performed in the foreseeable future. Simulation models are particularly helpful in this case to predict the long-term impact of interventions over time, account for uncertainties in real-world settings, and test various scenarios that may not be feasible to evaluate in traditional studies. By simulating different outcomes, we can provide more accurate predictions of both the health and economic benefits of interventions, offering valuable insights for decision-makers.

There is also a lack of research on the estimation of QALYs in bladder cancer. For example, the disutilities of both AC and NAC were not available from validated sources, such as RCTs. In the absence of reliable data, we used information about utilities from observational studies (9, 30, 31) and expert opinion (the information presented in **Supplementary Materials**). In addition to QALY at one-year follow-up, side effects emerging at even longer follow-up from cisplatin, an essential component in NAC, such as long-term renal impairment (32, 33), sensory neuropathy, tinnitus, and hearing loss (34) are well known and can also be taken into account. Thus, if a patient can be spared NAC based on molecular information, avoiding long-term side effects, there is another well-founded argument to implement this practice.

There are several limitations to be acknowledged in this study. First, our estimates of molecular subtype proportions, including the distribution of pathological outcomes in the cystectomy specimens stratifying patients into good and poor prognosis after RC, were derived from two small cohorts (3, 13). Due to the scarcity of sufficiently large cohorts with both molecular subtyping and clinical outcomes, our survival estimates for individuals with Ba/Sq tumors were retrieved in BladderBaSe 2.0, lacking subtype information (Section 1: **Supplementary Tables S1**-**S6**), although being a population-based source with real world data. The rationale for applying these data was based on similar survival outcomes reported for LundTax subtypes in patients treated with upfront RC in a Swedish population-based series (35) and in an observational multicenter study (36). Although our QALY estimates were based mainly on observational data and expert opinion, which might introduce bias, the baseline QALY estimation came from an RCT (18) and was based on Swedish tariff data. Thus, we have little reason to expect that this would introduce a bias when comparing NAC and AC. Another limitation was the use of only one classification system when assessing cost-effectiveness. Although the LundTax is well conformed with the MIBC consensus classification (37), several other molecular subtyping systems exist. Nonetheless, the Ba/Sq subtype defined by the MIBC consensus-classification system is also associated with chemoresistance in a recent RCT (4). Cross-comparison of various subtyping systems by clustering identified the Ba/Sq subtype as the only highly concordant across all systems, indicating that the choice of MIBC classifier matters less for the identification of this subtype. It is also likely that our outcomes are generalizable to gemcitabine-cisplatin as NAC regimen, as this cisplatin-based combination also was used in studies investigating the use of molecular subtypes as a measure to stratify the use of NAC (2–4). A limitation of our model is that adjuvant immune checkpoint inhibitors are not considered (38), even though a subtype dependent response to adjuvant checkpoint inhibitors has been suggested (39). This is beyond the scope of current study but highlights the need for future research when response probabilities on such therapy are available.

The reliability of a simulation model is contingent on the quality of the data and assumptions incorporated. Our cost data, sourced from Swedish registers, contributes to the internal validity of our findings. Nevertheless, external validity and cross-validity of a simulation model can be subject to scrutiny, although we conducted a range of sensitivity analyses to enhance the robustness of our results.

The low cost per QALY gained (€4,964/QALY) motivates the introduction of this strategy in Sweden and similar healthcare settings. This is also supported by the survival benefit from AC in patients with Ba/Sq tumors (40). In fact, a real-time population-based pipeline with prospective RNA-sequencing and molecular subtyping according to LundTax is already operating in several healthcare regions in Sweden (UROSCANSEQ, ISRCTN 15459149) (41), and a versatile and upgraded version of the LundTax classification algorithm applicable to different gene expression platforms and less sensitive to variations in sample purity is available (42). Furthermore, LundTax molecular subtyping can also be performed by immunohistochemistry on formalin-fixed specimens, further increasing the applicability of molecular classification in other healthcare contexts (43). A de-escalated subtype-based use of NAC might even increase the incentive to apply NAC in populations and countries where such preoperative treatment today is only used for a minority of patients, where overtreatment is the main objective against applying NAC. For example, only 21% of patients below 60 years of age received NAC in 2017 in Germany (44), and an even lower proportion in a report from the SEER data from the USA (17%) (45), despite being recommended to all eligible patients based on level 1a evidence (7).

## 5 Conclusions

Applying a de-escalated use of NAC in patients with MIBC with the Ba/Sq molecular subtype according to LundTax is cost-effective, and implementation in clinical practice can be considered.

## Data Availability

The data analyzed in this study is subject to the following licenses/restrictions: Data used for modeling is available upon request to the corresponding author (SS) and project leader (FL). Requests to access these datasets should be directed to sanjib.saha@med.lu.se.
