# Supplementary material for: Cost-effectiveness of de-escalated molecular subtype dependent use of neoadjuvant chemotherapy in patients with muscle-invasive bladder cancer in a Swedish setting
Source: Front Oncol. 2025 Apr 2;15:1556881. doi: 10.3389/fonc.2025.1556881 (PMC12000754; doi:10.3389/fonc.2025.1556881)
Supplement: Supplementary file 1 [file DataSheet1.docx]

Supplementary Material

## Section 1: Transitional probabilities used in the model

Table S1: Transitional probabilities of NAC+RC for current treatment procedure (n=553)

| Year | Survive | Dead by BC | Not dead by BC |
| --- | --- | --- | --- |
| 1 | 0.8702 | 0.1279 | 0.0019 |
| 2 | 0.7733 | 0.2227 | 0.004 |
| 3 | 0.7112 | 0.2822 | 0.0066 |
| 4 | 0.6721 | 0.3213 | 0.0066 |
| 5 | 0.6325 | 0.3565 | 0.011 |

Table S2: Transitional probabilities of NAC+RC for Gu/Uro subtype (n=77)

| Year | Survive | Dead by BC | Not dead by BC |
| --- | --- | --- | --- |
| 1 | 0.921 | 0.053 | 0.0263 |
| 2 | 0.790 | 0.184 | 0.0263 |
| 3 | 0.697 | 0.237 | 0.0658 |
| 4 | 0.658 | 0.276 | 0.0658 |
| 5 | 0.615 | 0.304 | 0.0804 |

Table S3: Transitional probabilities for RC only (current scenarios and for Ba/Sq subtype good prognosis) (n=1297)

| Year | Survive | Dead by BC | Not dead by BC |
| --- | --- | --- | --- |
| 1 | 0.7788 | 0.2163 | 0.0049 |
| 2 | 0.6542 | 0.3361 | 0.0097 |
| 3 | 0.5726 | 0.4139 | 0.0135 |
| 4 | 0.5273 | 0.4585 | 0.0142 |
| 5 | 0.4863 | 0.4952 | 0.0185 |

Table S4: Transitional probabilities RC+AC current scenarios and Ba/Sq subtypes with poor prognosis (n=73)

| Year | Survive | Dead by BC | Not dead by BC |
| --- | --- | --- | --- |
| 1 | 0.792 | 0.208 | 0 |
| 2 | 0.484 | 0.517 | 0 |
| 3 | 0.412 | 0.588 | 0 |
| 4 | 0.356 | 0.645 | 0 |
| 5 | 0.356 | 0.645 | 0 |

Table S5: Transitional probabilities Ba/Sq subtypes with good prognosis (Propensity score matched) (n=92)

| Year | Survive | Dead by BC | Not dead by BC |
| --- | --- | --- | --- |
| 1 | 0.7788 | 0.2163 | 0.0049 |
| 2 | 0.6542 | 0.3361 | 0.0097 |
| 3 | 0.5726 | 0.4139 | 0.0135 |
| 4 | 0.5273 | 0.4585 | 0.0142 |
| 5 | 0.4863 | 0.4952 | 0.0185 |

## Section 2: Estimation of Quality Adjusted Life Years (QALYs) for the model

### The concept of QALY

The quality-adjusted life-year (QALY) serves as a measure of the value of health outcomes. Given that health is influenced by both the length of life and the quality of life, the QALY was developed to combine the value of these attributes into a single index number. The QALY calculation involves multiplying the change in utility value induced by the treatment by the duration of the treatment effect to determine the number of QALYs gained or lost (Prieto and Sacristán 2003).

One widely used instruments for measuring QALY is the EQ-5D-5L questionnaire, a generic preferences-based measure that includes five attributes: mobility, self-care, usual activities, pain/discomfort, and anxiety/depression (Balestroni and Bertolotti 2012). Each attribute has five levels: no problems, slight problems, moderate problems, severe problems and extreme problems. These health states are translated into a score ranging from 0 (equivalent of dead) and 1 (perfect health) using a population preference value set known as a tariff. As population preferences vary from country to country, QALY estimation tariffs are country specific. The time spent in a health state is important for QALY estimation, and QALY is typically calculated for one year (365 days).

### Baseline line QALY estimation

The baseline QALY was derived from the iROC trial for participants who had not received NAC (Catto, Khetrapal, et al. 2022). All the patients provided responses to the five attributes of the EQ-5D-5L questionnaire, and the baseline QALY was subsequently estimated using the Swedish tariff (value set) (Burström et al. 2020). Despite the patients being from the UK, the decision to use the Swedish value set was based on the appropriateness for the Swedish population. The descriptive statistics are presented in Table 1.

Table S6: Descriptive statistics for the participants of the iROC trial (n=204)

| Measures | Values |
| --- | --- |
| Mean | 0.914 |
| Median | 0.943 |
| Standard deviation | 0.093 |
| Minimum | 0.372 |
| 5% percentile | 0.705 |
| 95% percentile | 0.9755 |
| Maximum | 0.9755 |

### *QALY for patients receiving only RC*

The postoperative QALY loss due to RC was 0.114 (0.80 QALY) (Joyce et al. 2022; Kulkarni et al. 2009). According to our assumptions, during the first 35 days, patients experience a QALY loss according to the iROC trial, with 50% of patients undergoing open RC and 50% robotic-assisted RC, as per current Swedish practice (SNRUBC 2022) (0.84 QALY). By day 84, QALY returns to 0.897 based on iROC data (Catto, Khetrapal, et al. 2022). Subsequently, we assumed utility returns to preoperative levels (0.914) at one year. Using the area under the curve method (Whitehead and Ali 2010) (see Figure S1), we have estimated the QALY for 365 days.

### *QALY for patients receiving three courses of NAC and RC*

The QALY reduction due to NAC was set to 0.36 QALY, in line with previous chemotherapy studies (Joyce et al. 2022; Kulkarni et al. 2009). This reduction corresponds to the administration of three courses of ddMVAC over 4 weeks, followed by RC 32 days later, based on the median time between the last chemotherapy course and RC in Sweden from 2020-2022 (RODRET)). On the day of RC, the baseline QALY for patients receiving NAC from the iROC trial was retrieved (0.909). The disutility for RC was 0.114 resulting in a postoperative QALY of 0.795. For upfront RC, we obtained QALY data from the iROC trial at 35 days in patients receiving NAC was obtained (0.833). Similarly, at day 84 after RC, QALY returns to 0.899 according to iROC data (Catto, Khetrapal, et al. 2022). Subsequently, we assumed that utility returns to preoperative levels (0.914) at one year. Using the area under the curve method (Whitehead and Ali 2010), we have estimated the QALY for 365 days (see Figure S2)

**

### *QALY estimation for patients receiving RC and four course of AC*

The postoperative QALY loss due to RC was 0.114 (0.80 QALY) based on studies by (Joyce et al. 2022; Kulkarni et al. 2009). For the initial 35 days, patients experience a QALY loss due to RC, as per the iROC trial (0.84 QALY). At day 84 day, the utility increased to 0.897. Following the approach from the largest AC trial in bladder cancer (Sternberg et al, lancet oncology 2015), where chemotherapy should be administered within 90 days of RC, we assumed that from day 85 and for an additional 42 days receiving AC with 4 courses of ddMVAC, the disutility was 0.36 (Kulkarni et al). After NAC, we estimate that QALY returns to baseline in 32 days after the last chemotherapy course (equal to day 156 after RC). For the remainder of the year, patients return to baseline QALY. The QALY for 365 days has been estimated using the area under the curve method (Whitehead and Ali 2010) (Figure S3).

## Section 3: Scenario analyses

## *First scenario analysis*

In our base case analysis, only 5.3% (n=73) patients received AC after RC. With information retrieved from the cystectomy specimen after RC on pathological tumour stage and nodal stage, where patients are both upstaged and down staged compared to their preoperative clinical tumour stage, we used information in BladderBaSe 2.0 on pathological tumour stage (available from 2008 and onwards). Thus, we estimated survival probabilities based on this information for patients with Ba/Sq subtype and good prognosis according to the cystectomy specimen (see Table 1 for definition) (n=191). To assume that these patients, where molecular subtypes are not known, represent survival probabilities in patients with Ba/Sq tumours is supported by the lack of prognostic information by molecular subtypes in patients treated with upfront RC (Kollberg et al. 2019).

## *Second scenario analysis*

Patients receiving NAC (n=553, Figure 1) generally have better physiological characteristics (e.g., younger, have less co-morbidities, better renal function etc.) than patients receiving RC only (n=1297, Figure 1). To adjust for known confounders, we used propensity score matching (PSM) to identify the match for the patients treated with NAC+RC (n=553) from the patients treated with RC only (n=1297) in the BladderBase 2.0 database. We used Probit regression, where variables included in the model were age, Charlson comorbidity index, clinical tumour stage, sex, educational status, grade, and healthcare region where the patients were treated based on known confounders (Table S7). Caliper matching with a width of 0.2 with no replacement and 1 to 1 matching method was used to identify the patients. We estimated survival probabilities among those as a proxy for patients with good prognosis according to the cystectomy specimen (n=92).

Table S7: The characteristics of the patients before and after propensity score matching

| Variables | Before Matching | | | After Matching | | |
| --- | --- | --- | --- | --- | --- | --- |
|  | RC (n=1297) | NAC+RC (n=553) | p value^*^ | RC (n=553) | NAC+RC  (n=553) | p value^*^ |
| Age | 66.7 (0.2) | 65.3 (0.3) | 0.00 | 65.2 (0.3) | 65.3 (0.3) | 0.91 |
| Charlson Comorbidity Index | 0.66 (0.03) | 0.47 (0.04) | 0.00 | 0.50 (0.04) | 0.47 (0.04) | 0.57 |
| Clinical Tumour Stage |  |  | 0.02 |  |  | 0.67 |
| -T2 | 1043 (80%) | 483 (87%) |  | 476 (86%) | 483 (87%) |  |
| -T3 | 189 (15%) | 51 (9%) |  | 53 (10%) | 51 (9%) |  |
| -T4 | 65 (5%) | 19 (3%) |  | 24 (4%) | 19 (3%) |  |
| Sex |  |  | 0.21 |  |  | 1.00 |
| -Men | 949 (73%) | 420 (76%) |  | 420 (76%) | 420 (76%) |  |
| -Women | 348 (27%) | 133 (24%) |  | 133 (23%) | 133 (24%) |  |
| Civil Status |  |  | 0.07 |  |  | 0.58 |
| -Unmarried/single | 173 (13%) | 94 (17%) |  | 92 (17%) | 94 (17%) |  |
| -Married/registered | 769 (59%) | 326 (59%) |  | 313 (57%) | 326 (59%) |  |
| -Divorced/widowed | 355 (27%) | 133 (24%) |  | 148 (27%) | 133 (24%) |  |
| Education |  |  | 0.01 |  |  | 0.99 |
| -Mandatory | 517 (40%) | 169 (31%) |  | 169 (31%) | 169 (31%) |  |
| -High school | 506 (39%) | 258 (47%) |  | 256 (46%) | 258 (47%) |  |
| -University | 265 (20%) | 124 (22%) |  | 126 (23%) | 124 (22%) |  |
| -Missing | 9 (1%) | 2 (0.4%) |  | 2 (0.4%) | 2 (0.4%) |  |
| Grade |  |  | 0.037 |  |  | 0.41 |
| -G1 and G2 | 126 (10%) | 43 (8%) |  | 32 (6%) | 43 (8%) |  |
| -G3 and GX | 1099 (85%) | 492 (89%) |  | 504 (91%) | 492 (89%) |  |
| -Missing | 72 (5%) | 18 (3%) |  | 17 (3%) | 18 (3%) |  |
| Healthcare Region |  |  | 0.000 |  |  | 0.95 |
| -Stockholm | 174 (13%) | 113 (20%) |  | 107 (19%) | 113 (20%) |  |
| -Southern | 279 (22%) | 116 (21%) |  | 110 (20%) | 116 (21%) |  |
| -Southeastern | 167 (13%) | 27 (5%) |  | 26 (5%) | 27 (5%) |  |
| -Uppsala/Örebro | 296 (23%) | 156 (28%) |  | 169 (31%) | 156 (28%) |  |
| -Western | 249 (19%) | 70 (13%) |  | 66 (12%) | 70 (13%) |  |
| -Northern | 132 (10%) | 71 (13%) |  | 75 (14%) | 71 (13%) |  |
| Year of diagnosis |  |  | 0.000 |  |  | 0.00 |
| -1999-2005 | 474 (37%) | 9 (2%) |  | 199 (36%) | 9 (2%) |  |
| -2006-2011 | 418 (32%) | 79 (14%) |  | 193 (35%) | 79 (14%) |  |
| -2012-2016 | 243 (19%) | 299 (54%) |  | 94 (17%) | 299 (54%) |  |
| -2017-2019 | 162 (12%) | 166 (30%) |  | 67 (12%) | 166 (30%) |  |

^*^T-test was used for the continuous variables and chi-square test was used for categorized variables

## *3.3 Third scenario analysis*

In our base case analysis, we excluded the costs and disutility associated with the three-course ddMVAC in the neoadjuvant situation and the four-course ddMVAC in the adjuvant condition. The primary reason for this was the insufficient studies identifying the prevalence of side effects following the three and four courses of ddMVAC for MIBC patients included in this study. The second reason was the insufficient research estimating the disutility and the toll associated with the side effects.
This scenario analysis utilized the study by Pilmack et al.(Plimack et al. 2014), which indicated that 12% experienced grade 3 or grade 4 adverse events following three doses of ddMAVC, whereas 18% experienced such toxicities after four courses of ddMVAC. The healthcare costs and the disutility associated with the side effects were derived from the Joyce et al. study (Joyce et al. 2022).

## *3.1 Result of the first scenario analysis*

Table S8. Incremental cost-effectiveness ratio (ICER) at 2-year, 3-year and 5-year by current treatment scenario and de-escalated molecular subtype-based use of perioperative chemotherapy, respectively

| Time duration | Cost (std) | Incremental cost | QALYs (std) | Incremental benefit | ICER (cost/QALY) |
| --- | --- | --- | --- | --- | --- |
| *2-year* | | | | | |
| Current treatment | 17,024 (1,189) |  | 2.20 (0.67) |  |  |
| Molecular subtype- | 17,934 (701) | 909 | 2.33 (0.59) | 0.13 | 6,865 |
| *3-year* | | | | | |
| Current treatment | 17,201 (1,222) |  | 2.56 (0.97) |  |  |
| Molecular subtype- | 18,166 (771) | 964 | 2.81 (0.89) | 0.24 | 3,937 |
| *5-year* | | | | | |
| Current treatment | 17,363 (1,287) |  | 2.89 (1.37) |  |  |
| Molecular subtype- | 18,405 (919) | 1,042 | 3.30 (1.38) | 0.41 | 2,572 |

Std=Standard deviation

## *Result of the second scenario analysis*

Table S9: Incremental cost-effectiveness ratio (ICER) at 2-year, 3-year and 5-year by current treatment scenario and de-escalated molecular subtype-based use of perioperative chemotherapy, respectively

| Time duration | Cost (Euro) (std) | Incremental cost | QALYs (std) | Incremental benefit | ICER (Euro/QALY) |
| --- | --- | --- | --- | --- | --- |
| *2-year* | | | | | |
| Current treatment | 17,011 (1,143) |  | 2.29 (0.63) |  |  |
| Molecular subtype- | 17,934 (669) | 932 | 2.35 (0.57) | 0.06 | 15,587 |
| *3-year* | | | | | |
| Current treatment | 17,230 (1,122) |  | 2.74 (0.93) |  |  |
| Molecular subtype- | 18,184 (715) | 954 | 2.85 (0.88) | 0.10 | 9,112 |
| *5-year* | | | | | |
| Current treatment | 17,459 (1,123) |  | 3.21 (1.42) |  |  |
| Molecular subtype- | 18,437  (839) | 978 | 3.37 (1.38) | 0.16 | 6,282 |

Std=Standard deviation

## *Result of the third scenario analysis*

Table S9: Incremental cost-effectiveness ratio (ICER) at 2-year, 3-year and 5-year by current treatment scenario and de-escalated molecular subtype-based use of perioperative chemotherapy, respectively

| Time duration | Cost (Euro) (std) | Incremental cost | QALYs (std) | Incremental benefit | ICER (Euro/QALY) |
| --- | --- | --- | --- | --- | --- |
| *2-year* | | | | | |
| Current treatment | 16,359 (1,635) |  | 2.16 (0.69) |  |  |
| Molecular subtype- | 18,874 (1,156) | 2,515 | 2.32 (0.58) | 0.17 | 15,225 |
| *3-year* | | | | | |
| Current treatment | 16,635 (1,876) |  | 2.53 (0.99) |  |  |
| Molecular subtype- | 19,404 (1,431) | 2,768 | 2.79 (0.89) | 0.26 | 10.463 |
| *5-year* | | | | | |
| Current treatment | 16,909 (2,215) |  | 2.88 (1.42) |  |  |
| Molecular subtype- | 19,940  (1,875) | 3,030 | 3.28 (1.37) | 0.40 | 7,641 |

Std=Standard deviation

**References**

Balestroni, Gianluigi, and Giorgio Bertolotti. 2012. 'EuroQol-5D (EQ-5D): an instrument for measuring quality of life', *Monaldi Archives for Chest Disease*, 78.

Burström, Kristina, Fitsum Sebsibe Teni, Ulf- G. Gerdtham, Reiner Leidl, Gert Helgesson, Ola Rolfson, and Martin Henriksson. 2020. 'Experience-Based Swedish TTO and VAS Value Sets for EQ-5D-5L Health States', *Pharmacoeconomics*, 38: 839-56.

Joyce, D. D., K. M. Wymer, V. Sharma, J. P. Moriarty, B. J. Borah, D. M. Geynisman, E. R. Plimack, B. A. Costello, L. C. Pagliaro, and S. A. Boorjian. 2022. 'Comparative cost-effectiveness of neoadjuvant chemotherapy regimens for muscle-invasive bladder cancer: Results according to VESPER data', *Cancer*, 128: 4194-202.

Kollberg, P., G. Chebil, P. Eriksson, G. Sjödahl, and F. Liedberg. 2019. 'Molecular subtypes applied to a population-based modern cystectomy series do not predict cancer-specific survival', *Urol Oncol*, 37: 791-99.

Kulkarni, G. S., S. M. Alibhai, A. Finelli, N. E. Fleshner, M. A. Jewett, S. R. Lopushinsky, and A. M. Bayoumi. 2009. 'Cost-effectiveness analysis of immediate radical cystectomy versus intravesical Bacillus Calmette-Guerin therapy for high-risk, high-grade (T1G3) bladder cancer', *Cancer*, 115: 5450-9.

Plimack, E. R., J. H. Hoffman-Censits, R. Viterbo, E. J. Trabulsi, E. A. Ross, R. E. Greenberg, D. Y. Chen, C. D. Lallas, Y. N. Wong, J. Lin, A. Kutikov, E. Dotan, T. A. Brennan, N. Palma, E. Dulaimi, R. Mehrazin, S. A. Boorjian, W. K. Kelly, R. G. Uzzo, and G. R. Hudes. 2014. 'Accelerated methotrexate, vinblastine, doxorubicin, and cisplatin is safe, effective, and efficient neoadjuvant treatment for muscle-invasive bladder cancer: results of a multicenter phase II study with molecular correlates of response and toxicity', *J Clin Oncol*, 32: 1895-901.

Prieto, L., and J. A. Sacristán. 2003. 'Problems and solutions in calculating quality-adjusted life years (QALYs)', *Health Qual Life Outcomes*, 1: 80.

SNRUBC. 2022. 'Svenska nationella kvalitetsregistret för Urinblåse- och urinvägscancer (SNRUBC) (The Swedish National Quality Register for Bladder and Urinary Tract Cancer)'. <https://statistik.incanet.se/Urinblasecancer/>.

Whitehead, Sarah J., and Shehzad Ali. 2010. 'Health outcomes in economic evaluation: the QALY and utilities', *British Medical Bulletin*, 96: 5-21.

**
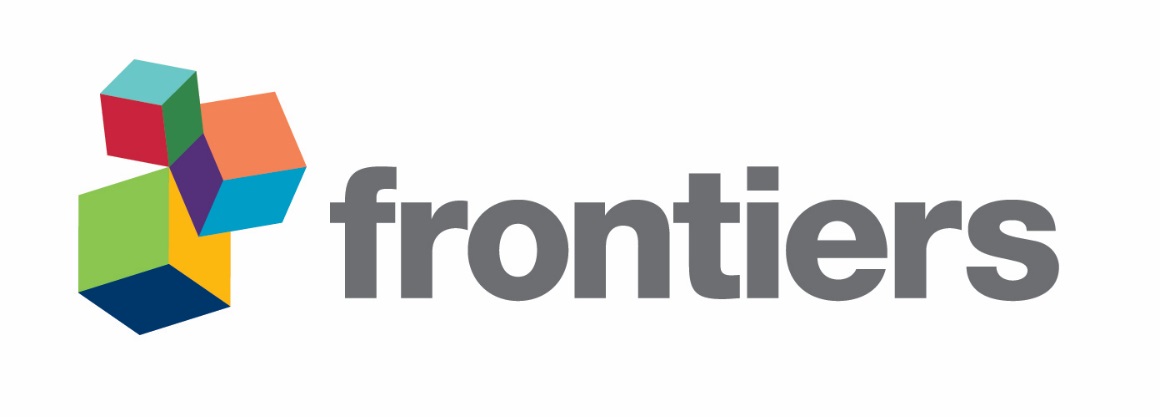
**
